# *Culex tarsalis* is a competent vector species for Cache Valley virus

**DOI:** 10.1186/s13071-018-3103-2

**Published:** 2018-09-20

**Authors:** Victoria B. Ayers, Yan-Jang S. Huang, Amy C. Lyons, So Lee Park, Stephen Higgs, James I. Dunlop, Alain Kohl, Barry W. Alto, Isik Unlu, Bradley J. Blitvich, Dana L. Vanlandingham

**Affiliations:** 1 Department of Diagnostic Medicine/Pathobiology, College of Veterinary Medicine, Kansas State University, Manhattan, KS 66506 USA; 2 Biosecurity Research Institute, Kansas State University, Manhattan, KS 66506 USA; 3 MRC-University of Glasgow Centre for Virus Research, Glasgow, G61 1QH Scotland, UK; 4 Florida Medical Entomology Laboratory, University of Florida, Vero Beach, FL 32962 USA; 5 Mercer County Mosquito Control, West Trenton, NJ 08628 USA; 6 Center for Vector Biology, Rutgers University, New Brunswick, NJ 08901 USA; 7 Department of Veterinary Microbiology and Preventive Medicine, College of Veterinary Medicine, Iowa State University, Ames, IA 50011 USA

**Keywords:** Cache Valley virus, Orthobunyavirus, Mosquito vectors, *Culex tarsalis*

## Abstract

**Background:**

Cache Valley virus (CVV) is a mosquito-borne orthobunyavirus endemic in North America. The virus is an important agricultural pathogen leading to abortion and embryonic lethality in ruminant species, especially sheep. The importance of CVV in human public health has recently increased because of the report of severe neurotropic diseases. However, mosquito species responsible for transmission of the virus to humans remain to be determined. In this study, vector competence of three *Culex* species mosquitoes of public health importance, *Culex pipiens*, *Cx. tarsalis* and *Cx. quinquefasciatus*, was determined in order to identify potential bridge vector species responsible for the transmission of CVV from viremic vertebrate hosts to humans.

**Results:**

Variation of susceptibility to CVV was observed among selected *Culex* species mosquitoes tested in this study. *Per os* infection resulted in the establishment of infection and dissemination in *Culex tarsalis*, whereas *Cx. pipiens* and *Cx. quinquefasciatus* were highly refractory to CVV. Detection of viral RNA in saliva collected from infected *Cx. tarsalis* provided evidence supporting its role as a competent vector.

**Conclusions:**

Our study provided further understanding of the transmission cycles of CVV and identifies *Cx. tarsalis* as a competent vector.

## Background

Cache Valley virus (CVV) is an orthobunyavirus endemic in North America [1–9]. Enzootic transmission of CVV occurs among ungulates through bites of competent arthropod vectors. Data from serological surveys and experimental infections indicate that white-tailed deer (*Odocoileus virginianus*) are likely to be the amplification hosts of the virus in nature [4–6, 8]. Historically, CVV has been regarded as an important agricultural pathogen in the USA but not a threat to people. Infection in adult sheep is common and results in recovery and seroconversion; however, infection during pregnancy often leads to embryonic and fetal death, stillbirths and multiple congenital malformations [10, 11]. The public health significance of CVV has been increasingly recognized because of recent reports of human diseases caused by infection of CVV and its variants. Four human cases of CVV infection have been diagnosed in the USA since 1995. In addition to the fatal case reported in North Carolina, neuroinvasion of CVV has been observed during the acute phase of the disease [12–15]. Seroprevalence rates among individuals with exposure to farm and wild animals in the USA were reported to exceed 3% [16]. A serological survey found that 5–7% of human serum samples, collected from two cities in Argentina, were positive for neutralizing antibodies [17]. It is possible that human infections in the Americas may be higher than indicated by the low number of symptomatic cases.

Whilst entomological surveys have been performed in the past, the objective of previously published studies was mainly to identify the species of enzootic vectors and their roles in the transmission and maintenance of CVV. It is well-accepted that multiple mosquito species in North America are competent for the transmission of CVV [2]. Six mosquito species have been demonstrated to be competent for CVV under laboratory conditions: *Culiseta inornata*, *Anopheles quadrimaculatus*, *Coquillettidia perturbans*, *Aedes sollicitans*, *Ae. taeniorhynchus* and *Ae. japonicus* [18–20]. In nature, virus isolations have been made in at least 16 mosquito species [2, 21]. However, very few studies have been performed to identify the species responsible for the transmission of CVV from amplification hosts to humans: so-called bridge vectors. Endemic vector species of CVV do not show host preference for humans. For example, *Cs. inornata*, one of the principle vector species in nature, does not normally feed on humans [22]. Similarly, populations of *An. quadrimaculatus* and *Cq. perturbans* have been shown to predominantly feed on non-human mammalian animals as observed with blood meal analyses conducted in several geographic regions [23–30]. Therefore, the zoonotic transmission of CVV in specific ecological conditions may involve other mosquito species that show host preference for both animals and humans as observed with multiple zoonotic arboviruses.

Although the percentage of CVV isolates obtained from *Culex* species mosquitoes is low in relation to the total number of available isolates, infection of CVV has been reported in at least three medically important species, *Cx. tarsalis*, *Cx. pipiens* and *Cx. restuans*, collected in the field. These observations warrant further investigation of whether or not *Culex* species mosquitoes can act as bridge vectors for the zoonotic transmission of CVV [2, 21, 31, 32]. The potential importance of North American *Culex* species mosquitoes for the transmission of zoonotic arboviruses to humans has been well-established for several viruses including St. Louis encephalitis virus (SLEV), Western equine encephalitis virus (WEEV), and West Nile virus (WNV) [33–35]. Therefore, determining the vector competence of medically important *Culex* species mosquitoes for CVV is likely to provide information on the vector species responsible for its transmission from viremic animals to humans. Vector competence based on orally challenged mosquitoes identifies species that are able to transmit CVV in nature and exclude the candidate vector species that became a source of viral isolation due to recent engorgement. In this study, three species of mosquitoes, *Cx. pipiens*, *Cx. quinquefasciatus* and *Cx. tarsalis*, were evaluated for their vector competence for CVV.

## Methods

### Cells and virus

African green monkey kidney epithelial Vero 76 cells were maintained in Leibovitz’s L-15 media (Thermo Fisher Scientific, Waltham, MA, USA) supplemented with 10% fetal bovine serum, 10% tryptose phosphate broth, penicillin/streptomycin, and L-glutamine, and used in this study for propagation of virus stocks and titration of homogenized tissues as previously described [36]. The prototype 6V633 strain of CVV was used in all oral challenge experiments for the determination of vector competence. It was originally isolated from infected *Cs. inornata* in Cache Valley, Utah, in 1956 [37]. Sequences of all three genomic segments have been determined in a previously published study (GenBank accession numbers: KX100133.1, KX100134.1 and KX100135.1) [38]. The strain was obtained from the collection in the laboratory of Dr Richard M. Elliot [39]. Stocks of CVV used in the oral infection study were generated by two passages in Vero 76 cells.

### Mosquitoes

Three medically important mosquitoes, *Cx. pipiens*, *Cx. tarsalis* and *Cx. quinquefasciatus*, were used in the experiments. Colonies of *Cx. pipiens* and *Cx. quinquefasciatus* were established from larvae collected in Ewing Township, New Jersey and Vero Beach, Florida, as previously described [40]. *Per os* infections of the two species were performed with F_8_ of *Cx. pipiens* and F_12_ of *Cx. quinquefasciatus*. *Cx. tarsalis* used in this study originated from a collection in Kern County, California [41]. The colonies were maintained by 10% sucrose solution under a 16:8 h light:dark photoregimen at 28 °C. Female mosquitoes aged 7–10 days-old were deprived of water and sucrose 24 and 48 h before *per os* infection, respectively. Viremic blood meals were prepared by mixing equal volumes of L-15 media that contained CVV at 7.95 log of 50% tissue culture infectious dose (TCID_50_)/ml with defibrinated sheep blood. Control mosquitoes received blood meals containing a 1:1 volume mixture of L-15 media and defibrinated sheep blood. Mosquitoes were allowed to orally ingest artificial infectious blood meals using previously published techniques [40, 42].

Engorged mosquitoes were collected under cold anesthesia and returned to designated cartons for characterization of the infection process. At 7 and 14 days post-infection (dpi), mosquitoes were collected and divided into two groups to characterize susceptibility to viral infection and dissemination in dissected mosquitoes and replication in whole carcasses. Infection status of each mosquito was determined by the isolation of infectious viruses using the TCID_50_-based titration method as previously described [36, 43]. Infection of individual mosquitoes was demonstrated by the detection of infectious viruses in dissected tissues or whole carcasses. Infection rates were calculated using the percentage of infected mosquitoes among all mosquitoes tested at each time-point. Disseminated form of infections were identified by the detection of infectious viruses in the secondary tissues of infected mosquitoes including the head, wings and legs. Dissemination rates were calculated by dividing the numbers of positive secondary tissues with the number of dissected mosquitoes that were infected with CVV. Growth kinetics of CVV in infected mosquitoes was determined based on the titers of CVV in whole mosquitoes. At 14 dpi, saliva from mosquitoes was collected to determine the incidence of transmission. Saliva was collected by inserting each mosquito’s proboscis into capillary tubes with type B immersion oil (Cargille Laboratories Inc., Cedar Grove, NJ, USA) for 1 h as previously described [36].

### Detection of CVV

Detection of CVV was performed by either the isolation of infectious viruses or the detection of viral genome. Quantities of infectious viruses in blood meals and homogenized mosquito tissues were determined with TCID_50_-based titration with Vero 76 cells as previously described [43]. Comparison of percentages of infection and dissemination was performed using Fisher’s exact test. The presence of viral genome in saliva of orally challenged mosquitoes was demonstrated by reverse-transcriptase polymerase chain reaction (RT-PCR). Extraction of viral RNA was performed with a QIAamp Viral RNA Mini Kit (Qiagen, Valencia, CA, USA). Viral RNA was reverse-transcribed with Superscript III Reverse Transcriptase (Invitrogen, Carlsbad, CA, USA). cDNA was amplified with a nested PCR approach based on previously published primer sets [44]. The outer primer set was designed to target nucleotide positions between 2220 and 2520 of the G1 gene encoded within the medium (M) genome segment. Amplicons derived from the outer primer set were amplified by the inner primer set targeting the nucleotide positions between 2246 and 2348 of the G1 gene.

## Results

### Infection and dissemination of CVV

Three species of medically important *Culex* species mosquitoes showed variations in susceptibility to CVV infection through oral exposure. As summarized in Table 1, the establishment of infection was only observed in *Cx. tarsalis*, whereas two species under the *Cx. pipiens* complex, *Cx. pipiens* and *Cx. quinquefasciatus*, were refractory to CVV. Infectious viruses of CVV were not detected among 28 and 27 *Cx. pipiens* collected at 7 and 14 dpi, respectively. Similarly, *Cx. quinquefasciatus* mosquitoes collected at 7 (*n* = 14) and 14 (*n* = 18) dpi did not show a detectable level of infectious viruses.

**Table 1:** Summary of infection and dissemination rates in *Culex* species mosquitoes orally challenged with Cache Valley virus

Mosquito species	7 dpi		14 dpi	
	Infection rate (%)^a^	Dissemination rate (%)^b^	Infection rate (%)^a^	Dissemination rate (%)^b^
*Cx. tarsalis*	81.8 (18/22)	72.7 (8/11)	82.6 (19/23)	100.0 (9/9)
*Cx. pipiens*	0.0 (0/28)	na	0.0 (0/27)	na
*Cx. quinquefasciatus*	0.0 (0/14)	na	0.0 (0/18)	na

*Abbreviation*: *na* not available

^a^Infection rates were derived from the percentage of infected mosquitoes among all the mosquitoes tested at each time-point (numbers in parentheses)

^b^Dissemination rates were calculated by dividing the numbers of mosquitoes containing positive secondary tissues with the number of dissected mosquitoes that were infected by CVV (numbers in parentheses)

**Fig. 1:**
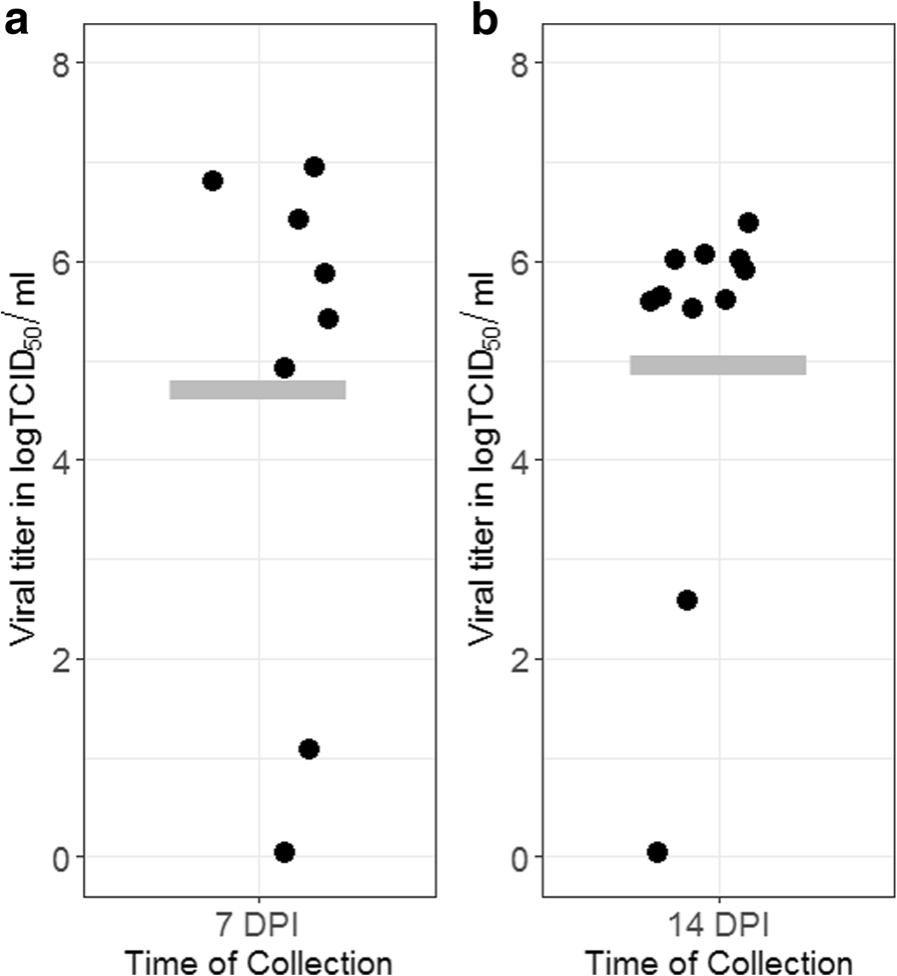
Viral titers of *Cx. tarsalis* infected with CVV at 7 (**a**) and 14 (**b**) days post-infection. The horizontal bar represents the average titer of whole mosquitoes

As demonstrated by the isolation of infectious viruses in homogenized mosquito tissues, there was no distinguishable difference in the infection rates of CVV in *Cx. tarsalis* at 7 (81.8%, 18/22) and 14 (82.6%, 19/23) dpi (*P* = 1.00). The dissemination rate of CVV in infected *Cx. tarsalis* showed a significant increase from 72.7% (8/11) at 7 dpi to 100.0% (9/9) at 14 dpi (Fisher’s exact test: *P* < 0.05), presumably due to the continuous viral replication in permissive tissue. However, there was no demonstrable difference in the average titer of infected whole mosquitoes at 7 (5.41 ± 2.06 logTCID_50_/ml, *n* = 7) and 14 (5.47 ± 1.07 logTCID_50_/ml, *n* = 10) dpi as shown in Fig. 1. Similarly, there were no significant differences in the infectious titers of CVV present in the dissected abdomen section (7 dpi: 4.23 ± 1.49 logTCID_50_/ml, *n* = 11; 14 dpi: 4.91 ± 0.51 logTCID_50_/ml, *n* = 8) and secondary tissues (7 dpi: 5.03±1.48 logTCID_50_/ml, *n* = 8; 14 dpi: 5.09 ± 1.48 logTCID_50_/ml, *n* = 9).

Detection of infectious viruses in mosquitoes collected at 7 and 14 dpi indicated that *Cx. tarsalis* is highly susceptible to CVV through oral challenge and subsequently supports viral replication. *Cx. pipiens* and *Cx. quinquefasciatus* are highly refractory to CVV.

### Detection of viral RNA in mosquito saliva

With the high infection and dissemination rates observed in *Cx. tarsalis* orally challenged with CVV, saliva obtained through forced salivation of individual mosquitoes at 14 dpi was assayed for the presence of the M segment of viral genome through nested RT-PCR. As anticipated, none of the saliva samples collected from *Cx. pipiens* (*n* = 27) and *Cx. quinquefasciatus* (*n* = 18) showed a detectable level of viral genome. Viral RNA of CVV was detected from 31.6% (6/19) of infected *Cx. tarsalis*. These results demonstrate that CVV is able to develop disseminated infection in *Cx. tarsalis*, which can subsequently be competent for its transmission.

## Discussion

The confirmation of *Cx. tarsalis* as a competent vector is of high public and veterinary health importance. Previous analyses of the blood-feeding behavior of *Cx. tarsalis* further supports its potential role in maintaining enzootic transmission of CVV, especially in the Midwestern states of the USA. In two independent studies, *Cx. tarsalis* from North Dakota and Minnesota, where CVV is endemic, showed relatively high frequencies of feeding on white-tailed deer, a known amplification host of CVV in nature [4, 45, 46]. In addition to its role as an enzootic vector, the established role of *Cx. tarsalis* as a vector species for SLEV, WNV and WEEV in western USA and its documented feeding on humans further supports the hypothesis that it may be involved in transmission of CVV from viremic animals to humans [34, 35]. Although there is variation in the frequency of feeding on humans, engorgement from human blood has been repeatedly observed in multiple populations of *Cx. tarsalis* in nature [29, 45–47]. Host preference is, in part, determined by changes in host availability, suggesting that the contact rate with humans may depend on the diversity of other potential hosts [48, 49].

As a species that has evolved to hibernate and has been shown to support the overwintering of arboviruses such as SLEV, WNV and WEEV [50, 51], our findings also highlight the need to further investigate the ecology of *Cx. tarsalis* and its involvement in the overwintering maintenance of CVV in nature. Similar to other orthobunyaviruses, vertical transmission has been demonstrated to be a likely overwintering mechanism for CVV [52]. For instance, 2.9 to 3.3% of experimentally infected *Cs. inornata* transovarially transmitted CVV to both male and female progeny. Further investigations in the detection of CVV in overwintering populations of *Cx. tarsalis* in nature will provide much needed understanding of the maintenance of CVV.

Whilst the number of reported neurotropic cases of CVV remains low, the advancement of virological and molecular biological techniques has led to the identification of variants or subtypes of CVV that are responsible for human diseases throughout the New World [53]. In 1985, the isolation of Fort Sherman virus was made from an American soldier in Panama who developed fever and an erythematous pharynx at the acute phase of infection [54]. Similarly, Maguari virus, another orthobunyavirus closely related to CVV, has been continuously found in multiple Latin American countries causing febrile illness in humans [38]. It remains unclear if other pathogenic orthobunyaviruses closely related to CVV can also utilize *Cx. tarsalis* or other medically important *Culex* species mosquitoes for transmission or maintenance. As observed with many other pathogenic arboviruses, identification of competent vector species can be an important step in formulating control strategies in the event of emergence.

## Conclusions

Since the original isolation of CVV in 1956, there have been isolates and detections of CVV made in *Culex* species mosquitoes [2, 21]. It has been unclear if the isolation or detection of CVV in *Culex* species mosquitoes was caused by recent engorgement of blood from viremic vertebrate hosts, which can cause a transient presence of infectious viruses and viral genomes in mosquitoes or actual viral replication, which is likely to result in a disseminated form of infection and transmission. To the best of our knowledge, this study provides the first direct evidence that *Cx. tarsalis* is susceptible to oral infection of CVV and competent for its transmission. Although we cannot exclude the possibility that other members in the *Cx. pipiens* complex can be competent for the transmission of CVV, the results of this study demonstrate that *Cx. pipiens* in New Jersey and *Cx. quinquefasciatus* in Florida, are highly refractory to CVV and are less likely to serve as competent vectors to support its transmission in nature.

## Abbreviations

CVV: Cache Valley virus
SLEV: St. Louis encephalitis virus
WEEV: Western equine encephalitis virus
WNV: West Nile virus
TCID_50_: 50% tissue culture infectious dose
dpi: Days post-infection
RT-PCR: Reverse-transcriptase polymerase chain reaction
M: Medium
